# Association between residual gastric volume and long-term postoperative sleep disturbances in gastric cancer survivors

**DOI:** 10.3389/fonc.2026.1798145

**Published:** 2026-04-15

**Authors:** Longxin Fan, Shaoying Li, Tao Meng, Sheng Zhang, Hailong Feng

**Affiliations:** Department of General Surgery, The First Affiliated Hospital of Xinxiang Medical University, Xinxiang, Henan, China

**Keywords:** gastric cancer, partial gastrectomy, Pittsburgh sleep quality index, residual gastric volume, sleep disturbance

## Abstract

**Background:**

Gastric cancer patients are frequently complicated by sleep disturbance, which severely impairs their quality of life and compromises disease prognosis. This study aims to investigate the association between residual gastric volume (RGV) and sleep disturbance in patients who have undergone partial gastrectomy, and to optimize clinical management.

**Methods:**

A total of 412 gastric cancer patients who underwent partial gastrectomy were enrolled. RGV was measured using ultrasonography at 6 months postoperatively; sleep disturbance was assessed with the Pittsburgh Sleep Quality Index (PSQI) at 6, 9, and 12 months postoperatively. Data regarding patients’ demographic characteristics, tumor features, treatment regimens, postoperative complications, dietary habits, and physical activity were collected via face-to-face interviews and medical record reviews. Multivariate logistic regression was performed to identify predictors of postoperative sleep disturbance, with adjustment for potential confounding factors.

**Results:**

Postoperative RGV ≤ 150 mL was associated with higher PSQI scores at 6, 9, and 12 months as well as a higher average total PSQI score, together with higher rates of frequent sleep symptoms (difficulty falling asleep, nocturnal awakening, early awakening, and daytime fatigue) during the 6–12-month follow-up (all P < 0.05).

**Conclusion:**

This study identifies a significant relationship between reduced RGV and persistent sleep disturbance, highlighting this gastric metric as a promising, reliable predictor of sleep-related complications in gastric cancer patients. If these findings are further validated, clinicians can identify high-risk populations for this complication at an early stage and implement precise prevention and management strategies through personalized dietary interventions and pharmacotherapy.

## 1 Introduction

According to the GLOBOCAN 2022 estimates, gastric cancer accounted for 968, 000 new cases and 660, 000 deaths worldwide in 2022 (1, 2); China alone contributed 359, 000 new cases and 260, 000 deaths, representing a substantial proportion of the global gastric cancer burden (1, 2). As a highly prevalent malignancy, gastric cancer not only severely impairs patients’ quality of life and shortens survival but also imposes a heavy burden on their families (3).

Previous studies have confirmed that approximately 62% to 86% of gastric cancer patients suffer from sleep disturbance, which has emerged as a critical factor affecting their physical and mental well-being (4, 5). Formulating intervention strategies targeting the known influencing factors constitutes an effective approach to improving sleep quality in these patients. Currently, in addition to the tumor itself, adverse marital status, low economic level, negative emotions, fatigue, and comorbid chronic diseases have all been associated with this complication (4–6). However, research on the specific gastric physiological factors influencing and predicting sleep disturbance remains limited.

Surgery is one of the primary treatment modalities for gastric cancer, and partial gastrectomy is indicated for the majority of patients with early-stage gastric cancer and no extensive metastasis (7). Residual gastric volume (RGV), a key and readily measurable postoperative physiological parameter, directly affects core gastric functions such as gastric emptying efficiency and eating tolerance. Studies have demonstrated that sleep quality improves concomitantly in obese patients following bariatric surgery and in patients with functional dyspepsia after the amelioration of gastric emptying function (8–10). Based on the current medical understanding of gastric physiology and existing research findings on the association between gastric structural and functional changes and sleep quality, we propose the following logical chain: RGV may induce nocturnal gastric discomfort by altering core gastric functions, ultimately impairing sleep quality. This hypothesis, however, remains to be validated in clinical studies.

Therefore, the present study adopted a prospective cohort design to systematically investigate the association between postoperative RGV and long-term sleep disturbance in gastric cancer patients. The study aimed to provide evidence-based support for identifying RGV as a predictive indicator of this complication and to offer a theoretical basis for optimizing postoperative sleep management strategies in gastric cancer patients.

## 2 Materials and methods

### 2.1 Ethics

This study was approved by the Institutional Review Board (IRB) of The First Affiliated Hospital of Xinxiang Medical University.

### 2.2 Study design and population

Within 10 large communities with uniform geographical distribution across Xinxiang City, Henan Province, and with the assistance of community physicians, newly diagnosed gastric cancer patients between January 1, 2018, and December 31, 2023, were screened. After community physicians contacted the patients and obtained their informed consent, they accompanied the researchers during home visits to complete enrollment registration based on pre-established inclusion and exclusion criteria, and to confirm valid contact information to facilitate subsequent research implementation.

Inclusion criteria: (1) Pathologically confirmed primary gastric cancer (11); (2) Underwent partial gastrectomy within 6 months prior to enrollment; preoperative or postoperative chemotherapy, radiotherapy, targeted therapy, or immunotherapy was permitted; (3) No evidence of tumor recurrence at enrollment (e.g., no recurrence indicated by imaging examinations or tumor markers); (4) No history of mental illness, cognitive disorders, sleep disturbance, or sleep-related diseases (e.g., obstructive sleep apnea) before gastric cancer diagnosis; (5) Stable chronic disease conditions (e.g., coronary heart disease, hypertension) from diagnosis to enrollment, without other severe comorbidities (e.g., other malignant tumors); (6) Voluntarily agreed to participate in this study (including follow-up for 6–12 months after surgery) and signed a written informed consent form.

Exclusion criteria: (1) Failure to meet any of the above inclusion criteria; (2) Confirmed gastric cancer recurrence during the follow-up period (6–12 months after surgery); (3) Loss to follow-up or active refusal of follow-up during the study period; (4) Development of other severe diseases or death during follow-up; (5) Inability to provide complete medical records (e.g., surgical records, pathological reports); (6) Inability to cooperate with postoperative RGV measurement or sleep disturbance assessment.

### 2.3 Data collection

At enrollment, the following information was collected through face-to-face interviews and medical record review:

(1) Preoperative baseline characteristics of participants, including demographic information, medical history, prior treatment history, and family medical history. Detailed items are listed in **Table 1**, with definitions of medical history variables provided in the notes to **Table 1**.

**Table 1:** Basic characteristics of the subjects before surgery.

Variable	SRGV	MLRGV	t/χ²	P
Total (n)	178 (100.0)	234 (100.0)	—	—
Demographic information				
Age at enrollment (year)	62.4 ± 9.2	61.2 ± 11.1	1.136	0.257
Male (n)	112 (62.9)	136 (58.1)	0.973	0.324
Non-married (n)	51 (28.7)	54 (23.1)	1.654	0.198
No fixed income (n)	38 (21.3)	41 (17.5)	0.955	0.328
No medical insurance (n)	43 (24.2)	52 (22.2)	0.213	0.644
Personal history				
Smoking (n)	72 (40.4)	84 (35.9)	0.890	0.345
Drinking (n)	68 (38.2)	75 (32.1)	1.688	0.194
Pickled/grilled food intake (n)	82 (46.1)	94 (40.2)	1.436	0.231
Lack of vegetable/fruit intake (n)	103 (57.9)	122 (52.1)	1.338	0.247
Irregular meal times (n)	115 (64.6)	137 (58.5)	1.563	0.211
High-salt diet (n)	91 (51.1)	108 (46.2)	1.000	0.317
Lack of physical activity (n)	105 (59.0)	126 (53.8)	1.085	0.297
Disease history				
Obesity/overweight (n)	64 (36.0)	71 (30.3)	1.446	0.229
Type 2 diabetes (n)	44 (24.7)	45 (19.2)	1.798	0.180
Hypertension (n)	78 (43.8)	92 (39.3)	0.846	0.358
Coronary heart disease (n)	27 (15.2)	30 (12.8)	0.468	0.494
Cerebrovascular disease (n)	17 (9.6)	19 (8.1)	0.260	0.610
Gastric ulcer (n)	43 (24.2)	38 (16.2)	4.013	0.045
Chronic atrophic gastritis (n)	80 (44.9)	82 (35.0)	4.154	0.042
Hp infection (n)	113 (63.5)	127 (54.3)	3.526	0.060
Previous treatment history				
Proton pump inhibitor usage (n)	76 (42.7)	88 (37.6)	1.093	0.296
Partial gastrectomy (n)	9 (5.1)	6 (2.6)	1.790	0.181
Family history				
Gastric cancer (n)	25 (14.0)	27 (11.5)	0.576	0.448

SRGV, Small residual gastric volume; MLRGV, Medium/large residual gastric volume; Hp, Helicobacter pylori. Smoking: ≥1 cigarette per week; Drinking: ≥4 drinks per week (1 drink ≈14g alcohol); Pickled/grilled food intake: ≥3 times per week, ≥100g each time; Lack of vegetable/fruit intake: Total daily intake of vegetables and fruits <500g, ≥5 days per week; Irregular meal times: ≥4 days per week with missed breakfast, dinner after 19:00, or interval between meals >6 hours; High-salt diet: Daily salt intake ≥6g (including condiments); Lack of physical activity: Weekly moderate-intensity exercise <150 minutes or high-intensity exercise <75 minutes. All above personal histories refer to a duration of ≥1 year within 10 years. Continuous variables were expressed as mean ± standard deviation. Categorical variables were expressed as frequency and constituent ratio. A value of P < 0.05 indicated statistical significance.

(2) Gastric cancer-related characteristics, including pathological features, preoperative treatment, surgical interventions, in-hospital complications, and postoperative treatment. Detailed items are presented in **Table 2**.

**Table 2:** Gastric cancer-related characteristics and RGVs.

Variable	SRGV	MLRGV	t/χ²	P
Total (n)	178 (100.0)	234 (100.0)	—	—
Pathological characteristics				
Tumor differentiation (n)				
Well-moderately	81 (45.5)	129 (55.1)	3.746	0.053
Poorly-undifferentiated	97 (54.5)	105 (44.9)		
TNM stage (n)				
stage I	35 (19.7)	64 (27.4)	3.273	0.070
stage II-III	143 (80.3)	170 (72.6)		
Tumor size >5cm (n)	74 (41.6)	79 (33.8)	2.643	0.104
Vascular invasion (n)	59 (33.1)	56 (23.9)	4.266	0.039
Nerve invasion (n)	57 (32.0)	53 (22.6)	4.538	0.033
Preoperative treatment				
Chemotherapy (n)	34 (19.1)	36 (15.4)	0.990	0.320
Radiotherapy (n)	17 (9.6)	18 (7.7)	0.449	0.503
Targeted/immunotherapy (n)	8 (4.5)	6 (2.6)	1.148	0.284
Surgical treatment				
Surgical approach (n)				
Laparoscopic surgery	112 (62.9)	163 (69.7)	2.067	0.150
Open surgery	66 (37.1)	71 (30.3)		
Gastrectomy scope (n)				
Proximal gastrectomy	46 (25.8)	72 (30.8)	1.201	0.273
Distal gastrectomy	132 (74.2)	162 (69.2)		
Lymph node dissection scope (n)				
D1-D1+	34 (19.1)	62 (26.5)	3.093	0.079
D2	144 (80.9)	172 (73.5)		
Anastomosis method (n)				
Billroth I	39 (21.9)	72 (30.8)	4.031	0.045
Billroth II	51 (28.7)	73 (31.2)	0.311	0.577
Roux-en-Y anastomosis	88 (49.4)	89 (38.0)	5.365	0.021
Blood loss >200 ml (n)	65 (36.5)	67 (28.6)	2.886	0.089
Surgical duration >180 min (n)	78 (43.8)	81 (34.6)	3.614	0.057
In-hospital complications				
Nausea and vomiting (n)	28 (15.7)	26 (11.1)	1.894	0.169
Wound infection (n)	15 (8.4)	11 (4.7)	2.374	0.123
Postoperative ileus (n)	13 (7.3)	9 (3.8)	2.391	0.122
Intra-abdominal infection (n)	10 (5.6)	7 (3.0)	1.763	0.184
Postoperative bleeding (n)	8 (4.5)	4 (1.7)	2.773	0.096
Anastomotic leakage (n)	6 (3.4)	2 (0.9)	3.361	0.067
Postoperative treatment				
Chemotherapy (n)	93 (52.2)	110 (47.0)	1.110	0.292
Radiotherapy (n)	26 (14.6)	27 (11.5)	0.849	0.357
Targeted/immunotherapy (n)	10 (5.6)	8 (3.4)	1.170	0.279
At 6 months after surgery				
Postoperative RGV (ml)	113.7 ± 20.9	223.7 ± 42.7	31.618	<0.001

RGV, Residual gastric volume, SRGV, Small residual gastric volume; MLRGV, Medium/large residual gastric volume; TNM, Tumor Node Metastasis. Continuous variables were expressed as mean ± standard deviation. Categorical variables were expressed as frequency and constituent ratio. A value of P < 0.05 indicated statistical significance.

### 2.4 RGV measurement

At 6 months postoperatively, a home visit was conducted to measure RGV using a Mindray M5 portable ultrasound system. Following an 8-hour fast, the operator identified the residual stomach via ultrasound scanning and calculated RGV using the ellipsoid volume formula; three repeated measurements were obtained to ensure accuracy (12).

### 2.5 Sleep and psychological assessment

At 6, 9, and 12 months postoperatively, home visits were conducted to assess sleep disturbance, depression, anxiety, and quality of life using validated standardized scales: the Pittsburgh Sleep Quality Index (PSQI) for sleep disturbance, the Patient Health Questionnaire-9 (PHQ-9) for depression, the Generalized Anxiety Disorder-7 (GAD-7) for anxiety, and the 36-Item Short Form Health Survey (SF-36) for quality of life.

The total PSQI score ranges from 0 to 21, with higher scores indicating poorer sleep quality; a score > 7 is commonly used to define clinically significant sleep disturbance (13). The PHQ-9 score ranges from 0 to 27, with higher scores reflecting more severe depressive symptoms (14). The GAD-7 score ranges from 0 to 21, with higher scores indicating greater anxiety severity (15). The SF-36 yields domain scores from 0 to 100 for each dimension, with higher scores representing better health-related quality of life (16).

### 2.6 Follow-up

At 6, 9, and 12 months postoperatively, post-discharge complications, post-discharge treatments, dietary patterns, physical activity, and nutritional status were documented during home visits; detailed items are listed in **Tables 3**, **4**. Definitions of selected post-discharge complications are provided in the notes to **Table 3**. Nutritional status was assessed using body mass index (BMI), peripheral hemoglobin, and albumin measured at the three follow-up time points, with data extracted from routine follow-up medical records.

**Table 3:** Complications and corresponding treatments from 6 months to 12 months after surgery.

Variable	SRGV	MLRGV	t/χ²	P
Total (n)	178 (100.0)	234 (100.0)	—	—
Post-discharge complications				
Dyspepsia (n)	51 (28.7)	57 (24.4)	0.963	0.326
Gastroesophageal reflux (n)	47 (26.4)	51 (21.8)	1.185	0.276
Diarrhea (n)	31 (17.4)	29 (12.4)	2.050	0.152
Malabsorption (n)	31 (17.4)	27 (11.5)	2.887	0.089
Anastomotic stenosis (n)	18 (10.1)	16 (6.8)	1.432	0.231
Post-discharge treatments				
Nutritional supplements (n)	68 (38.2)	72 (30.8)	2.490	0.115
Proton pump inhibitors (n)	48 (27.0)	44 (18.8)	3.884	0.049
Gastric mucosal protectants (n)	46 (25.8)	43 (18.4)	3.328	0.068
Digestive enzymes (n)	41 (23.0)	45 (19.2)	0.885	0.347
Prokinetic agents (n)	39 (21.9)	41 (17.5)	1.244	0.265
Antidiarrheals (n)	23 (12.9)	20 (8.5)	2.069	0.150

SRGV, Small residual gastric volume; MLRGV, Medium/large residual gastric volume. Dyspepsia: Symptoms such as abdominal distension and early satiety caused by impaired digestive function of the residual stomach after surgery (lasting ≥ 1 week). Malabsorption: Insufficient nutrient absorption within 6 months after surgery, manifested as weight loss ≥ 5% or anemia, etc. Post-discharge treatments: Refers to regular use of medications for relieving postoperative complications, with a duration of ≥ 30 days after discharge. Continuous variables were expressed as mean ± standard deviation. Categorical variables were expressed as frequency and constituent ratio. A value of P < 0.05 indicated statistical significance.

**Table 4:** Dietary patterns, physical activities, and nutritional status from 6 months to 12 months after surgery.

Variable	SRGV	MLRGV	t/χ²	P
Total (n)	178 (100.0)	234 (100.0)	—	—
Dietary patterns				
Meal frequency (n)				
3 times/day	19 (10.7)	41 (17.5)	3.809	0.051
4–5 times/day	113 (63.5)	145 (62.0)	0.099	0.753
≥6 times/day	46 (25.8)	48 (20.5)	1.631	0.202
Food intake (n)				
<100g/meal	94 (52.8)	105 (44.9)	2.551	0.110
≥100g/meal	84 (47.2)	129 (55.1)		
Predominant staple food (n)				
Rice/noodles	94 (52.8)	147 (62.8)	4.174	0.041
Porridge/soup	84 (47.2)	87 (37.2)		
Eating 2 hours before bed (n)	33 (18.5)	58 (24.8)	2.293	0.130
Overheated food intake (n)	28 (15.7)	46 (19.7)	1.058	0.304
ONS intake (n)	68 (38.2)	72 (30.8)	2.490	0.115
Physical activity				
Frequency of exercise (n)				
0–1 time/week	64 (36.0)	61 (26.1)	4.676	0.031
2 times/week	85 (47.8)	119 (50.9)	0.389	0.533
≥3 times/week	29 (16.3)	54 (23.1)	2.893	0.089
Duration of exercise (n)				
<30 minutes	75 (42.1)	82 (35.0)	2.156	0.142
30–60 minutes	81 (45.5)	113 (48.3)	0.315	0.575
≥60 minutes	22 (12.4)	39 (16.7)	1.487	0.223
Type of exercise (n)				
Walking	129 (72.5)	151 (64.5)	2.929	0.087
Jogging/running	31 (17.4)	53 (22.6)	1.706	0.191
Strength training	18 (10.1)	30 (12.8)	0.720	0.396
Nutritional status				
Average BMI (kg/m^2^)	21.7 ± 2.0	22.1 ± 2.4	2.114	0.035
Average hemoglobin (g/l)	119.9 ± 11.6	121.6 ± 11.4	1.536	0.125
Average albumin (g/l)	35.9 ± 3.4	36.8 ± 4.1	2.311	0.021

SRGV, Small residual gastric volume; MLRGV, Medium/large residual gastric volume; ONS, Oral nutritional supplement; BMI, Body mass index. Continuous variables were expressed as mean ± standard deviation. Categorical variables were expressed as frequency and constituent ratio. A value of P < 0.05 indicated statistical significance.

In addition, data on hypnotic medication use were collected; relevant items and definitions are presented in **Table 5** and its corresponding note.

**Table 5:** Sleep quality, depression, anxiety, and quality of life from 6 months to 12 months after surgery.

Variable	SRGV	MLRGV	t/χ²	P
Total (n)	178 (100.0)	234 (100.0)	—	—
Sleep assessment				
PSQI score at 6 months	8.0 ± 2.5	7.5 ± 2.1	2.524	0.012
PSQI score at 9 months	8.2 ± 2.5	7.5 ± 2.0	2.840	0.005
PSQI score at 12 months	8.2 ± 2.5	7.7 ± 2.0	2.208	0.028
Average PSQI total score	8.1 ± 1.4	7.6 ± 1.1	4.573	<0.001
Difficulty falling asleep (n)				
≥1 episode/week (Frequently)	72 (40.4)	69 (29.5)	5.397	0.020
<1 episode/week (Rarely)	106 (59.6)	165 (70.5)		
Nocturnal awakening (n)				
≥1 episode/week (Frequently)	77 (43.3)	74 (31.6)	5.894	0.015
<1 episode/week (Rarely)	101 (56.7)	160 (68.4)		
Early awakening (n)				
≥1 episode/week (Frequently)	63 (35.4)	56 (23.9)	6.466	0.011
<1 episode/week (Rarely)	115 (64.6)	178 (76.1)		
Daytime fatigue (n)				
≥1 episode/week (Frequently)	82 (46.1)	73 (31.2)	9.527	0.002
<1 episode/week (Rarely)	96 (53.9)	161 (68.8)		
Hypnotic medications				
Benzodiazepines (n)	34 (19.1)	35 (15.0)	1.245	0.264
Non-benzodiazepines (n)	38 (21.3)	41 (17.5)	0.955	0.328
Other assessment				
Average PHQ-9 score	9.0 ± 2.5	8.7 ± 1.8	1.554	0.121
Average GAD-7 score	6.5 ± 2.2	6.1 ± 1.9	1.678	0.094
Average SF-36 score	56.1 ± 12.2	59.1 ± 14.5	2.220	0.027

SRGV, Small residual gastric volume; MLRGV, Medium/large residual gastric volume; PSQI, Pittsburgh sleep quality index; PHQ-9, Patient health questionnaire-9; GAD-7, Generalized anxiety disorder-7; SF-36 = 36-Item short form health survey. Difficulty falling asleep: Time from preparing for sleep to falling asleep exceeds 30 minutes. Nocturnal awakening: Waking up ≥1 time during nighttime sleep, with difficulty falling back asleep within 15 minutes. Early awakening: Waking up ≥60 minutes earlier than the planned wake-up time, and unable to fall asleep again. Daytime fatigue: Persistent tiredness, lack of energy, or drowsiness during daytime activities, affecting daily functioning. All means were calculated from the measurements obtained at 6, 9, and 12 months after surgery. Hypnotic medications: Regular use for ≥30 days to improve sleep disturbance after discharge; Benzodiazepines mainly included estazolam, Non-benzodiazepines mainly included zolpidem tartrate tablets in this study. Continuous variables were expressed as mean ± standard deviation. Categorical variables were expressed as frequency and constituent ratio. A value of P < 0.05 indicated statistical significance.

### 2.7 Statistical analysis

Continuous variables were expressed as mean ± standard deviation. Categorical variables were expressed as frequencies and percentages. Between-group differences were evaluated using the independent samples t-test and chi-square test, respectively.

Multivariate logistic regression analysis was performed to identify factors associated with postoperative sleep disturbance, by examining the relationship between postoperative RGV and sleep quality, depression, anxiety, and quality of life at 6 to 12 months after surgery, with adjustment for potential confounding factors.

Potential covariates were initially selected from variables in **Tables 1**-**4**, including preoperative baseline characteristics, gastric cancer-related features, postoperative complications and treatments, and dietary, nutritional, and physical activity variables. First, variables with a between-group difference of P < 0.1 were defined as potential RGV−related variables. Next, all patients were stratified into two groups according to the average PSQI total score: a score ≤ 7 indicated normal sleep quality, and a score > 7 indicated sleep disturbance. Between-group differences in these potential RGV−related variables were then tested according to sleep quality. Only variables with P < 0.1 for both associations — i.e., related to both RGV and sleep disturbance — were included as covariates in the final multivariate logistic regression model (17, 18). Collinearity diagnostics were performed using the variance inflation factor (VIF); a VIF < 10 indicated no significant multicollinearity.

Mediation analysis was further conducted using linear regression with the bootstrap method (1000 replications) to examine the mediating effects of BMI and albumin on the association between postoperative RGV and sleep disturbance (defined as average PSQI total score>7), with adjustment for the same covariates as in the logistic regression model.

A P-value < 0.05 was considered statistically significant. All analyses were performed using SPSS 29.0.

*Post hoc* power analysis was conducted using GPowerWin_3.1.9.7 (19).

## 3 Results

### 3.1 Subjects

A total of 432 subjects met the initial inclusion criteria and were enrolled in this study. During the follow-up period, 12 withdrew due to developing other severe diseases, 5 due to failure to provide complete medical records, and 3 due to voluntary refusal to continue participation, resulting in a final cohort of 412 patients (95.4%) who completed the entire study.

Referring to previous research, investigators have used 110 mL as the cutoff value for RGV stratification when exploring the impact of remnant gastric volume on quality of life in patients after distal gastrectomy for gastric cancer (20). Considering the distribution characteristics of the measured RGV data at 6 months postoperatively in the current study, 150 mL was selected as the cutoff to ensure balanced sample sizes and sufficient statistical power between the two groups: 178 subjects with RGV ≤ 150 mL were assigned to the small RGV (SRGV) group, and 234 subjects with RGV > 150 mL were allocated to the medium/large RGV (MLRGV) group.

### 3.2 Basic characteristics of the subjects before surgery

In **Table 1**, compared with the MLRGV group, the SRGV group had significantly higher proportions of patients with gastric ulcer and chronic atrophic gastritis (P = 0.045 and P = 0.042, respectively). No statistically significant intergroup differences were observed in the other variables, indicating that the baseline characteristics of the participants were well balanced.

### 3.3 Gastric cancer-related characteristics and RGVs

In **Table 2**, as the grouping variable, the RGV at 6 months postoperatively was significantly lower in the SRGV group than in the MLRGV group (113.7 ± 20.9 ml, 223.7 ± 42.7 ml, P < 0.001).

Furthermore, compared with the MLRGV group, the SRGV group had higher proportions of patients with vascular invasion and nerve invasion (P = 0.039 and P = 0.033, respectively). Additionally, the SRGV group showed a lower proportion of patients who underwent Billroth I anastomosis (P = 0.045) but a higher proportion of those who underwent Roux-en-Y anastomosis (P = 0.021) compared with the MLRGV group. Although gastric cancer-related characteristics in the SRGV group appeared to indicate more advanced disease, with patients receiving more aggressive treatment and having a higher incidence of postoperative complications, no statistically significant intergroup differences were found for most indicators. This suggests that the gastric cancer−related characteristics were reasonably well balanced between the two groups.

### 3.4 Complications and corresponding treatments from 6 months to 12 months after surgery

In **Table 3**, compared with the MLRGV group, the SRGV group had a significantly higher proportion of patients receiving proton pump inhibitor therapy after discharge (P = 0.049). Furthermore, although the data indicated that patients in the SRGV group had a greater variety of post-discharge complications and received more additional post-discharge treatments, none of these indicators exhibited statistically significant intergroup differences. This demonstrated that the distribution of post-discharge complications was well balanced between the two groups.

### 3.5 Dietary patterns, nutritional status, and physical activities from 6 months to 12 months after surgery

In **Table 4**, compared with the MLRGV group, the SRGV group had a higher proportion of patients whose predominant staple food was porridge/soup (P = 0.041), a higher percentage of individuals with less frequent exercise (0–1 time/week) (P = 0.031), and significantly lower levels of average BMI and average albumin (P = 0.035 and P = 0.021, respectively). Most other indicators showed no statistically significant differences between groups, suggesting that dietary patterns, nutritional status, and physical activities were adequately balanced across groups during follow-up.

### 3.6 Sleep quality, depression, anxiety, and quality of life from 6 months to 12 months after surgery

In **Table 5**, **Supplementary Figure 1**, compared with the MLRGV group, the SRGV group had significantly higher PSQI scores at 6 months, 9 months, and 12 months postoperatively, as well as a higher average total PSQI score (P = 0.012, P = 0.005, P = 0.028, and P < 0.001, respectively). The SRGV group showed higher proportions of patients reporting frequent sleep symptoms (≥ 1 time/week), including difficulty falling asleep, nocturnal awakening, early awakening, and daytime fatigue (P = 0.020, P = 0.015, P = 0.011, and P = 0.002, respectively). Additionally, there was no statistically significant intergroup difference in the hypnotic medication use between the two groups (P = 0.264, P = 0.328, respectively).

Furthermore, the average SF-36 score was significantly lower in the SRGV group than in the MLRGV group (P = 0.027), while no significant differences were observed between the two groups in terms of average PHQ-9 and GAD-7 scores (P = 0.121, P = 0.094, respectively).

### 3.7 Relationships between potential RGV-related variables and sleep quality

Variables with potential between-group differences (P < 0.1) in **Tables 1**–**4** were defined as potential RGV-related variables and included in the following analyses to evaluate their association with sleep quality (average PSQI total score).

In **Supplementary Table 1**, compared with patients who had an average PSQI total score of ≤ 7, those with an average PSQI total score of>7 had significantly higher proportions of chronic atrophic gastritis and Hp infection (P = 0.013, P = 0.048, respectively), as well as higher proportions of TNM stage II-III, vascular invasion and nerve invasion (P = 0.025, P = 0.006, P = 0.003). They also had a significantly higher proportion of Roux-en-Y anastomosis (P = 0.020) but a markedly lower proportion of Billroth I anastomosis (P < 0.001), along with a higher proportion of post-discharge malabsorption (P = 0.013), a higher proportion of meal frequency 4–5 times per day (P = 0.070), and a lower proportion of meal frequency ≥ 6 times per day (P = 0.009). In addition, the proportions of patients who took rice/noodles as the predominant staple food, exercised 0–1 time per week and chose walking as the main type of exercise were significantly higher in the group with an average PSQI total score of>7 (P = 0.023, P < 0.001, P < 0.001), while their average BMI and average albumin levels were significantly lower (both P < 0.001).

Therefore, these variables were potentially associated with both RGV and sleep quality (average PSQI total score) (all P<0.1).

### 3.8 Association of postoperative RGV with sleep quality, depression, anxiety, and quality of life from 6 months to 12 months after surgery

**Figure 1:**
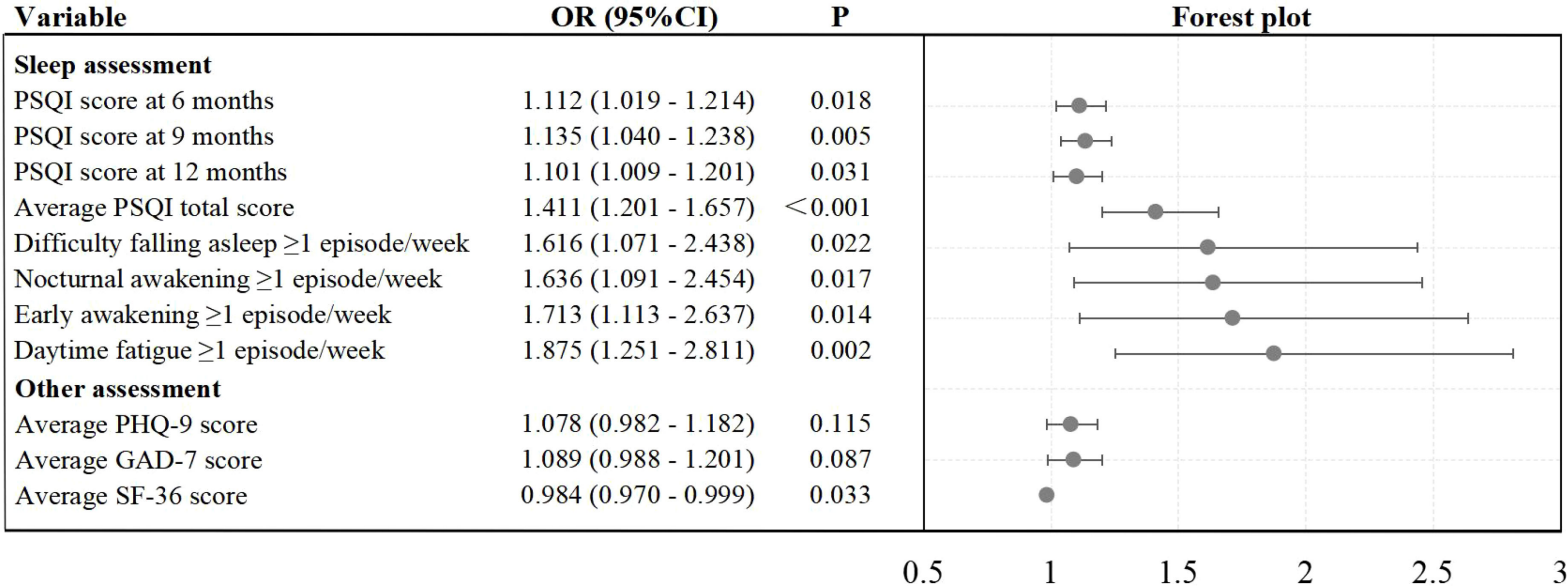
Association of postoperative RGV with sleep quality, depression, anxiety, and quality of life from 6 months to 12 months after surgery. Note: OR, Odds ratio; 95%CI, 95% Confidence interval; PSQI, Pittsburgh sleep quality index; PHQ-9, Patient health questionnaire-9; GAD-7, Generalized anxiety disorder-7; SF-36 = 36-Item short form health survey, RGV, Residual gastric volume; OR, Odds ratio; 95%CI, 95% Confidence interval. Solid circles represented the ORs and horizontal bars denoted the 95% CIs. The multivariate model was adjusted for disease history (chronic atrophic gastritis, Hp infection), TNM stage, vascular invasion, nerve invasion, anastomosis method, post-discharge malabsorption, meal frequency, predominant staple food, exercise frequency, exercise type, average BMI, and average albumin. A value of P < 0.05 indicated statistical significance.

Combining the results from **Tables 1**-**4**, **Supplementary Table 1**, only variables that were potentially associated with both RGV and sleep quality (average PSQI total score) were included as covariates in the multivariate logistic regression model. This model was used to evaluate the associations of postoperative RGV with sleep quality, depression, anxiety, and quality of life during follow-up, aiming to identify predictors of postoperative sleep disturbance. The VIF values for all covariates were < 10, indicating no substantial multicollinearity. Specific covariates are listed in the footnote of **Figure 1**.

In **Figure 1**, the multivariate logistic regression model showed that postoperative SRGV was associated with higher PSQI scores at 6 months, 9 months, and 12 months, as well as a higher average total PSQI score, during the 6- to 12-month follow-up period (P = 0.018, P = 0.005, P = 0.031, and P < 0.001, respectively). Additionally, postoperative SRGV was associated with higher proportions of frequent sleep symptoms (≥ 1 episode/week) over the same period, including difficulty falling asleep, nocturnal awakening, early awakening, and daytime fatigue (P = 0.022, P = 0.017, P = 0.014, and P = 0.002, respectively). These association findings suggested that postoperative SRGV might be an independent predictor of sleep disturbance, although further validation was warranted.

Furthermore, postoperative SRGV was associated with a lower average SF-36 score during the 6- to 12-month period (P = 0.033), but was not significantly related to the average PHQ-9 or GAD-7 scores (P = 0.115, P = 0.087, respectively).

### 3.9 Mediation effects of BMI and albumin on the association between postoperative RGV and sleep disturbance

In **Supplementary Table 2**, neither BMI nor albumin exerted a significant mediating effect on the association between postoperative RGV and sleep disturbance (indirect effect: P = 0.425, P = 0.540, respectively). These results indicated that these two nutritional indicators were not key mediators between postoperative RGV and sleep disturbance.

### 3.10 Power analysis

A *post-hoc* power analysis (G*Power 3.1.9.7) for the key association (postoperative SRGV vs. average total PSQI score) in **Figure 1** showed an achieved power of 94.8%. The analysis was based on an observed effect size of Cohen’s f²=0.0374, total sample size of 412 patients, and α=0.05 (two-tailed). This confirmed sufficient statistical power for the study.

## 4 Discussion

To the best of our knowledge, this is the first study to focus on this research domain in gastric cancer survivors following partial gastrectomy. We centered our investigation on RGV for two primary reasons. First, prior studies have exclusively explored the impact of abnormal gastric emptying function on sleep quality, with no research to date addressing the relevance of gastric structural characteristics in this context. Second, RGV holds greater practical value in clinical and community practice: postoperative RGV in patients stabilizes over time, can objectively reflect the anatomical properties of the residual stomach after surgery, and is easy to measure. In contrast, gastrointestinal function-related indicators are susceptible to multiple confounding factors and typically require repeated measurements at specific time points. Furthermore, RGV and gastric emptying function indicators are not mutually independent; RGV can, to a certain extent, influence and reflect gastric emptying efficiency as well as gastric content retention status.

We specifically opted to measure RGV at 6 months postoperatively to avoid the confounding effects of short-term factors associated with the surgical recovery period. We additionally set the 6–12-month postoperative window as our observation period, which balances the need for repeated assessments of sleep quality with patient follow-up compliance. Furthermore, the low risk of tumor recurrence during this phase eliminates potential interference from disease progression, enabling a more precise analysis of the association between postoperative RGV and long-term sleep disturbance in gastric cancer patients and, in turn, facilitating the identification of predictors for sleep disturbance.

This study demonstrated that an SRGV at 6 months postoperatively was associated with a 10%-40% increase in PSQI scores at three time points and the average score during the 6-12-month follow-up period. It was also linked to a 60%-90% higher risk of frequent sleep symptoms (≥1 episode/week) during this period, including difficulty falling asleep, nocturnal awakening, early awakening, and daytime fatigue. Consistent results were observed across PSQI scores (both individual time points and the average) and key sleep symptom outcomes, indicating good stability of the findings.

Clinically, a smaller RGV after gastrectomy for gastric cancer is generally considered to be associated with more extensive surgical resection and more advanced disease. Our study demonstrated a similar trend between the two groups, although most of the relevant comparisons did not reach statistical significance (**Tables 1**-**4**), suggesting acceptable baseline balance between groups.

For confounding factors that showed statistically significant differences (P<0.05) or marginally significant differences (0.05≤P<0.1) in association with both RGV and sleep disturbance and might interfere with the primary study results, the present study performed adjustments using a multivariate logistic regression model designed to identify independent predictors of postoperative sleep disturbance. This covariate selection approach, combined with VIF analysis, enabled precise control of potential confounding effects and enhanced the independence and reliability of the study findings.

Additionally, the inclusion criteria of this study excluded patients with mental illnesses, cognitive disorders, sleep-related diseases prior to gastric cancer diagnosis, as well as those with unstable chronic diseases, further strengthening the robustness of our findings.

In addition, preliminary mediation analysis failed to confirm the mediating role of nutritional factors including BMI and albumin in the association between postoperative RGV and sleep disturbance, which does not completely rule out the potential involvement of nutritional factors but merely indicates that the underlying mechanism is multifactorial and complex, and warrants further comprehensive research to explore all potential pathways, including an in-depth investigation into nutritional-related mechanisms, thereby enabling the development of more targeted interventions.

The present study also found that an SRGV at 6 months postoperatively was associated with reduced quality of life (assessed by the average SF-36 score) during the 6-12-month follow-up period, which is consistent with previous research (6). However, it was not correlated with concurrent depression and anxiety levels (evaluated by the average PHQ-9 and GAD-7 scores), which is inconsistent with prior findings (5, 6). This discrepancy may be explained by the more direct impact of SRGV on quality of life, whereas the development of depression and anxiety is regulated by multiple factors such as psychosocial status and perceived disease prognosis, which may have weakened the association between SRGV and these emotional outcomes.

The potential association between SRGV and sleep disturbance may be mediated through multiple pathophysiological pathways. On one hand, abnormal RGV can directly induce gastrointestinal motility disorders, leading to uncomfortable symptoms such as nocturnal gastroesophageal reflux. These nocturnal somatic discomforts can repeatedly disrupt sleep cycles and reduce sleep continuity and depth (21). On the other hand, gastrointestinal dysfunction can impair the sleep-wake rhythm of the central nervous system by disrupting the regulation of the brain-gut axis (22), and may be accompanied by intestinal flora imbalance, which indirectly exacerbates the disruption of sleep architecture (23). In addition, long-term gastrointestinal discomfort can trigger chronic somatic stress, leading to dysregulated secretion of stress hormones and further aggravating the development of sleep disturbance (24, 25).

## 5 Limitations

This study has several limitations. First, although power analysis confirmed that the sample size and statistical power of this study met statistical requirements, the single-center study design may compromise sample representativeness, thereby limiting the generalizability of the study findings. Second, this study only measured RGV and did not conduct objective, comprehensive assessments of other key gastric function indicators such as gastric emptying rate and gastrointestinal motility, which hinders an in-depth exploration of the intrinsic association between gastric physiological changes and sleep disturbance. Third, sleep disturbance was evaluated solely using the PSQI, a subjective self-reported scale, with no supplementary objective sleep monitoring metrics (e.g., polysomnography and sleep latency tests) incorporated, which may introduce potential bias into the assessment of actual sleep quality. Fourth, this study only verified a significant clinical correlation between RGV and sleep disturbance, and no in-depth mechanistic research was performed to elucidate the specific pathophysiological pathways and regulatory factors underlying this association. These limitations are mainly attributable to practical/feasibility constraints.

Based on these limitations, multicenter, large-sample cohort studies will be prioritized in future research to further validate the predictive value of RGV for postoperative sleep disturbance in gastric cancer patients, with a key focus on investigating the synergistic predictive value of RGV and gastric emptying function indicators. This will provide a quantitative basis for the more accurate clinical screening of gastric cancer patients at high risk of postoperative sleep disturbance. Meanwhile, mechanistic research can be conducted in combination with research directions such as brain-gut axis regulation and intestinal flora imbalance to clarify the specific pathophysiological pathways linking RGV and sleep disturbance. In addition, prospective interventional studies can be designed based on relevant research results to verify the clinical efficacy of dietary and pharmaceutical interventions targeting the improvement of residual gastric physiological status in the prevention and management of postoperative sleep disturbance, thereby providing feasible clinical strategies for sleep management in gastric cancer patients after surgery.

## 6 Conclusion

This study confirms a significant association between reduced postoperative RGV and persistent sleep disturbance in patients with gastric cancer. As a readily accessible clinical gastric metric, RGV holds promising potential to serve as a reliable predictor of sleep-related complications in this patient population. If the findings of this study are further validated by future research, targeted screening and intervention strategies could be implemented in clinical practice: RGV assessment may be incorporated into routine follow-up examinations for gastric cancer patients at 3 to 6 months postoperatively, enabling early prediction and risk stratification of postoperative sleep disturbance; meanwhile, personalized dietary regimens tailored to the physiological characteristics of the residual stomach may be formulated for patients, with scientific adjustments to meal frequency, portion sizes and food types, and adjunctive treatment with gastric emptying-promoting medications administered when necessary. These measures would allow for the precise prevention and management of postoperative sleep disturbance at the source, thereby effectively improving the postoperative quality of life for gastric cancer survivors.

## Data Availability

The raw data supporting the conclusions of this article will be made available by the authors, without undue reservation.
