# Supplementary material for: Association between residual gastric volume and long-term postoperative sleep disturbances in gastric cancer survivors
Source: Front Oncol. 2026 Apr 15;16:1798145. doi: 10.3389/fonc.2026.1798145 (PMC13124568; doi:10.3389/fonc.2026.1798145)
Supplement: Supplementary file 1 [file DataSheet1.docx]

**Supplemental Table 1 Relationships between potential RGV-related variables and sleep quality stratified by average PSQI total score**

| **Variable** | **Average PSQI total score＞7** | **Average PSQI total score≤7** | **t / χ²** | **P** |
| --- | --- | --- | --- | --- |
| Total (n) | 317 (100.0) | 95 (100.0) | － | － |
| **Disease history** |  |  |  |  |
| Gastric ulcer (n) | 67 (21.1) | 14 (14.7) | 1.895 | 0.169 |
| Chronic atrophic gastritis (n) | 135 (42.6) | 27 (28.4) | 6.148 | 0.013 |
| Hp infection (n) | 193 (60.9) | 47 (49.5) | 3.913 | 0.048 |
| **Pathological characteristics** |  |  |  |  |
| Tumor differentiation (n) |  |  |  |  |
| Well-moderately | 156 (49.2) | 54 (56.8) | 1.703 | 0.192 |
| Poorly-undifferentiated | 161 (50.8) | 41 (43.2) |  |  |
| TNM stage (n) |  |  |  |  |
| stage I | 68 (21.5) | 31 (32.6) | 5.005 | 0.025 |
| stage II-Ⅲ | 249 (78.5) | 64 (67.4) |  |  |
| Vascular invasion (n) | 99 (31.2) | 16 (16.8) | 7.520 | 0.006 |
| Nerve invasion (n) | 96 (30.3) | 14 (14.7) | 9.028 | 0.003 |
| **Surgical treatment** |  |  |  |  |
| Lymph node dissection scope (n) |  |  |  |  |
| D1-D1+ | 68 (21.5) | 28 (29.5) | 2.632 | 0.105 |
| D2 | 249 (78.5) | 67 (70.5) |  |  |
| Anastomosis method (n) |  |  |  |  |
| Billroth Ⅰ | 70 (22.1) | 41 (43.2) | 16.495 | ＜0.001 |
| Billroth Ⅱ | 101 (31.9) | 23 (24.2) | 2.034 | 0.154 |
| Roux-en-Y anastomosis | 146 (46.1) | 31 (32.6) | 5.376 | 0.020 |
| Blood loss >200 ml (n) | 106 (33.4) | 26 (27.4) | 1.237 | 0.266 |
| Surgical duration >180 min (n) | 127 (40.1) | 32 (33.7) | 1.255 | 0.263 |
| **In-hospital complications** |  |  |  |  |
| Postoperative bleeding (n) | 10 (3.2) | 2 (2.1) | 0.741 | 0.451 |
| Anastomotic leakage (n) | 7 (2.2) | 1 (1.1) | 0.688 | 0.415 |
| **Post-discharge complications** |  |  |  |  |
| Malabsorption (n) | 52 (16.4) | 6 (6.3) | 6.150 | 0.013 |
| **Post-discharge treatments** |  |  |  |  |
| Proton pump inhibitors (n) | 75 (23.7) | 17 (17.9) | 1.400 | 0.237 |
| Gastric mucosal protectants (n) | 74 (23.3) | 15 (15.8) | 2.463 | 0.117 |
| **Dietary patterns** |  |  |  |  |
| Meal frequency (n) |  |  |  |  |
| 3 times/day | 48 (15.1) | 12 (12.6) | 0.370 | 0.543 |
| 4-5 times/day | 206 (65.0) | 52 (54.7) | 3.279 | 0.070 |
| ≥6 times/day | 63 (19.9) | 31 (32.6) | 6.756 | 0.009 |
| Predominant staple food (n) |  |  |  |  |
| Rice/noodles | 195 (61.5) | 46 (48.4) | 5.161 | 0.023 |
| Porridge/soup | 122 (38.5) | 49 (51.6) |  |  |
| **Physical activity** |  |  |  |  |
| Frequency of exercise (n) |  |  |  |  |
| 0-1 time/week | 112 (35.3) | 13 (13.7) | 16.206 | ＜0.001 |
| 2 times/week | 159 (50.2) | 45 (47.4) | 0.227 | 0.633 |
| ≥3 times/week | 46 (14.5) | 37 (38.9) | 27.132 | ＜0.001 |
| Type of exercise (n) |  |  |  |  |
| Walking | 239 (75.4) | 41 (43.2) | 34.885 | ＜0.001 |
| Jogging/running | 62 (19.6) | 22 (23.2) | 0.583 | 0.445 |
| Strength training | 16 (5.0) | 32 (33.7) | 58.236 | ＜0.001 |
| **Nutritional status** |  |  |  |  |
| Average BMI (kg/m^2^) | 21.5±2.1 | 23.3±1.9 | 7.635 | ＜0.001 |
| Average albumin (g/l) | 35.6±3.7 | 39.0±3.2 | 8.228 | ＜0.001 |

Note: RGV = Residual gastric volume, PSQI = Pittsburgh sleep quality index, BMI = Body mass index. All patients were stratified into two groups according to the average PSQI total score: a score of＞7 indicated sleep disturbance, and a score of ≤ 7 indicated normal sleep quality. Continuous variables were expressed as mean±standard deviation. Categorical variables were expressed as frequency and constituent ratio. A value of P < 0.05 indicated statistical significance.

**Supplementary Table 2 Mediation analysis of BMI and albumin in the association between postoperative RGV and sleep disturbances (average PSQI total score)**

| **Mediator /**  **Effect type** | **β** | **95%CI** | **P** |
| --- | --- | --- | --- |
| Total effect (Postoperative RGV on sleep disturbance) | 0.411 | 0.201 - 0.657 | ＜0.001 |
| **BMI** |  |  |  |
| Path a (Postoperative RGV on BMI) | 0.040 | 0.002 - 0.078 | 0.035 |
| Path b (BMI on sleep disturbance, adjusted for RGV) | -0.025 | -0.086 - 0.036 | 0.421 |
| Direct effect (Postoperative RGV on sleep disturbance, adjusted for BMI) | 0.412 | 0.200 - 0.660 | ＜0.001 |
| Indirect effect (Postoperative RGV via BMI on sleep disturbance) | -0.001 | -0.005 - 0.003 | 0.425 |
| **Albumin** |  |  |  |
| Path a (Postoperative RGV on albumin) | 0.090 | 0.014 - 0.166 | 0.021 |
| Path b (Albumin on sleep disturbance, adjusted for RGV) | -0.018 | -0.075 - 0.039 | 0.538 |
| Direct effect (Postoperative RGV on sleep disturbance, adjusted for albumin) | 0.413 | 0.202 - 0.661 | ＜0.001 |
| Indirect effect (Postoperative RGV via albumin on sleep disturbance) | -0.002 | -0.008 - 0.004 | 0.540 |

Note: RGV = Residual gastric volume, PSQI = Pittsburgh sleep quality index, BMI = Body mass index, 95%CI = 95% Confidence interval, SRGV = Small residual gastric volume, MLRGV = Medium/large residual gastric volume. Mediation analysis was conducted using the bootstrap method with 1000 replications; an indirect effect was considered statistically significant if the 95%CI did not contain 0. The dependent variable was the average PSQI total score (continuous), the independent variable was postoperative RGV (dichotomized as SRGV/MLRGV), and BMI/albumin were set as separate mediating variables, respectively. All regression models were adjusted for the same covariates as the multivariate logistic regression in Figure 1. Total effect = Direct effect + Indirect effect; the indirect effect represents the mediating effect of the corresponding nutritional indicator in the association between postoperative RGV and sleep disturbance.


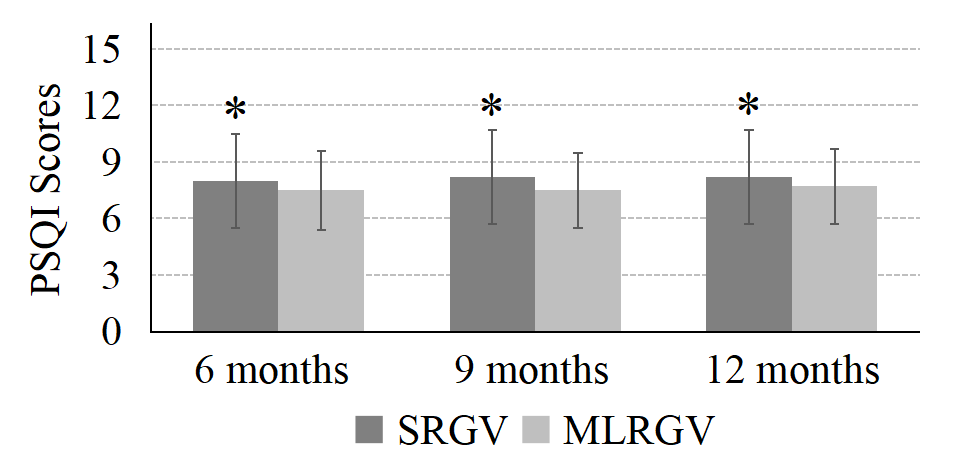


**Supplemental Figure 1 Sleep quality (PSQI Scores) in SRGV and MLRGV groups at 6, 9 and 12 months after surgery**

Note: SRGV = Small residual gastric volume, MLRGV = Medium/large residual gastric volume, PSQI = Pittsburgh Sleep Quality Index.

The height of each bar represents the mean value, and the error bars indicate the standard deviation. Asterisks indicate a significant difference at P < 0.05 vs. the MLRGV group.
